# Prevalence of Cognitive Impairment and Mental Health Disorders in Peripheral Arterial Disease Patients: Implications for Surgical Outcomes

**DOI:** 10.1192/bjo.2025.10200

**Published:** 2025-06-20

**Authors:** Marianne Bergman, Henry Bergman, Andrew Busuttil, Hesham Abdelkhalek, Ellie White

**Affiliations:** ^1^Essex Partnership Foundation Trust, Wickford, United Kingdom; ^2^Imperial College London, London, United Kingdom; ^3^Cambridge University Hospitals, Cambridge, United Kingdom; ^4^University of Cambridge, Cambridge, United Kingdom

## Abstract

**Aims:** Peripheral Arterial Disease (PAD) is linked to vascular cognitive impairment and vascular depression due to concurrent cerebrovascular disease. Cognitive impairment and mental health disorders in surgical patients lead to poorer recovery and rehabilitation. Awareness of these diagnoses in this population is important when considering suitability for surgical input – especially high-risk procedures such as limb revascularisation. In the UK population, evidence suggests that 33–42% of cognitive impairment and 26.5% of anxiety and depressive disorders remain undiagnosed. In this high-risk PAD population, this is likely an underestimate. We aim to determine the prevalence of cognitive impairment and anxiety and depressive disorders within this group while controlling for possible confounders.

**Methods:** 100 unselected PAD patients were identified pragmatically from a Vascular outpatient setting at Addenbrooke’s Hospital. Inclusion criteria required patients to be >65, and have radiological or clinical evidence of PAD. Written consent was obtained for assessment. Patients came from a wide area in the East of England with a catchment of 1.8 million people, demonstrating significant socio-geographical variety for a single centre study. The assessment pack included the 6-CIT screening tool for cognitive impairment, HADS for anxiety and depression, 4AT for delirium, SASQ for alcohol use, and a brief clinical history covering memory, attention, visuospatial skills, speech, and personality.

**Results:** 100 patients were assessed over 6 months. 20.5% met the 6-CIT cut-off for memory clinic referral (mean score 12). The estimated general prevalence of cognitive impairment including dementia in those over 65 is 7.1%. 26.8% met the HADS cut-off for anxiety or depression (mean score 15). The prevalence of anxiety and depression in the general over 65 population is estimated to be around 9% and 12.5%, respectively. Hence, there appears to be a significant increase in possible prevalence of both diagnoses, compared with a general age matched population. No significant age or gender differences were found between those scoring above the cut offs and those not (p=0.79).

**Conclusion:** Cognitive impairment and anxiety/depression appear more prevalent in PAD patients. Given these conditions impact surgical outcomes, raising awareness of underdiagnoses is crucial. Future follow up will include memory clinic assessment, cerebral and peripheral imaging, and exploring surgical outcomes. Cost-effectiveness analyses are needed to assess the benefits of pre-operative screening.

